# Infection image: cystic pneumocystis jirovecii pneumonia – forgotten?

**DOI:** 10.1007/s15010-024-02403-w

**Published:** 2024-10-14

**Authors:** Florian Hitzenbichler, Christoph Fisser, Alexandra Schlitt, Bernd Salzberger

**Affiliations:** 1 Department of Infection Prevention and Infectious Diseases, University Hospital Regensburg, Franz- Josef-Strauß-Allee 11, 93053 Regensburg, Germany; 2 Department of Internal Medicine II, University Hospital Regensburg, Franz-Josef-Strauß-Allee 11, 93053 Regensburg, Germany; 3 Department of Radiology, University Hospital Regensburg, Franz-Josef-Strauß-Allee 11, 93053 Regensburg, Germany

## Abstract

A 43-year-old male patient presented to the emergency department with progressive dyspnea. CT scan showed pronounced cystic lesions and ground glass opacitiy in both lungs and diagnosis of HIV infection was established. Bronchoscopy confirmed diagnosis of pneumocystis jirovecii pneumonia (PCP). The radiological presentation with perihilar large cysts is typical for PCP in HIV-infected patients, but rarely encountered today.

A 43-year-old male patient presented to the emergency department with dyspnea. The symptoms persisted after a SARS-CoV2 infection three months earlier. He reported a previously positive HIV test, but had not sought treatment yet. He was stable with an oxygen saturation of 93% while breathing 2 L O2 (via nasal cannula). The CT scan revealed predominantly perihilar cystic lesions, consolidation and ground glass opacity (GGO) in both lungs (Fig 1 A and C).

Serological testing confirmed HIV infection with a viral load of 1.1 × 10^6 cop/ml, CD4 count was 12/µl. A presumptive diagnosis of pneumocystis jirovecii pneumonia (PCP) was made, cotrimoxazol was started with additional prednisolone. Bronchoscopy confirmed PCP (via PCR). Other infections were excluded (tuberculosis, aspergillosis, cryptococcus, SARS-CoV-2, influenza, CMV, etc.).

Antiretroviral therapy was started within few days. After a 3-week course cotrimoxazol was continued as a prophylactic regime thrice weekly. The patient recovered and a CT scan three months later revealed significant improvement of all cystic lung lesions (Fig 1 B and D).

This radiological presentation, the cystic form of PCP, is rarely observed today, however, it is typical in patients with HIV-infection. In patients with other forms of immunosuppression (e.g. organ transplantation) GGO and septal thickening is more frequently seen [1–3].

With PCP as a typical opportunistic infection in HIV late presenters, knowledge of classical radiological findings is crucial for establishing the diagnosis. Pathogenesis of developments of cysts is poorly understood, but normally cystic lesions resolve with therapy and immune reconstitution [4].

**Fig. 1:**
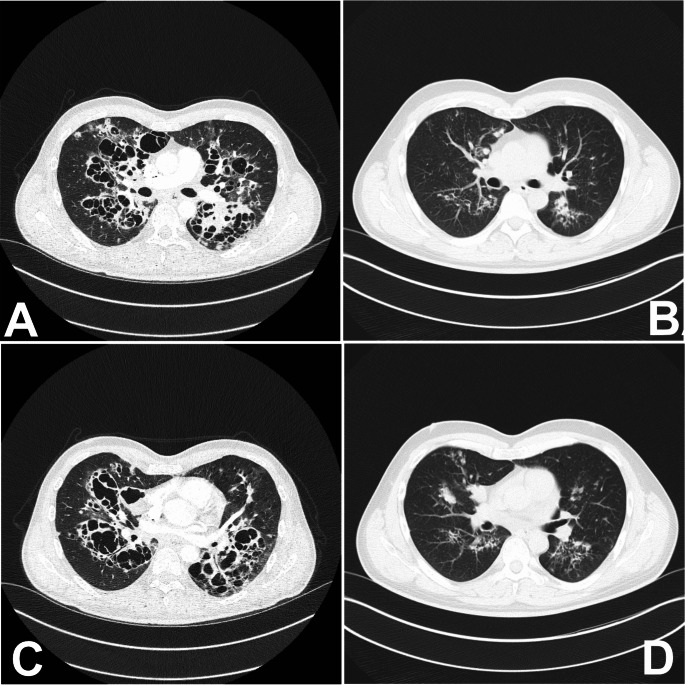
Figure 1 A&C: Radiological image (CT scan) at presentation Figure 1 B&D: Radiological image after three months

## Data Availability

No datasets were generated or analysed during the current study.

## References

[1] Ebner L, Walti LN, Rauch A, Furrer H, Cusini A, Meyer AM, et al. Clinical course, Radiological manifestations, and outcome of Pneumocystis Jirovecii Pneumonia in HIV patients and renal transplant recipients. PLoS ONE. 2016;11(11):e0164320. 10.1371/journal.pone.0164320. 27824870 10.1371/journal.pone.0164320 PMC5100884

[2] Fätkenheuer G, Franzen C, Hartmann P, Wassermann K, Schrappe M, Dölken W, et al. [Cystic form of Pneumocystis carinii pneumonia]. Dtsch Med Wochenschr. 1997;122(47):1441–6. 10.1055/s-2008-1047783. 9424421 10.1055/s-2008-1047783

[3] Salzer HJF, Schäfer G, Hoenigl M, Günther G, Hoffmann C, Kalsdorf B, et al. Clinical, diagnostic, and treatment disparities between HIV-Infected and Non-HIV-Infected immunocompromised patients with Pneumocystis Jirovecii Pneumonia. Respiration. 2018;96(1):52–65. 10.1159/000487713. 29635251 10.1159/000487713

[4] Chow C, Templeton PA, White CS. Lung cysts associated with pneumocystis carinii pneumonia: radiographic characteristics, natural history, and complications. AJR Am J Roentgenol. 1993;161(3):527–31. 10.2214/ajr.161.3.8352098. 8352098 10.2214/ajr.161.3.8352098

